# Sociodemographic factors and their predictive role in parents’ future anxiety

**DOI:** 10.1186/s41155-025-00340-7

**Published:** 2025-03-17

**Authors:** Anna M. Bujnowska, Celestino Rodríguez, Trinidad García, Débora Areces, Nigel V. Marsh

**Affiliations:** 1 Department of Psychology, University of Oviedo (Spain), Plaza Feijoo, CP: 33003 Oviedo, Asturias Spain; 2 Tropical Futures Institute, James Cook University, Singapore, Singapore

**Keywords:** Parents, Future anxiety, Families, Parental educational level, Future anxiety scale

## Abstract

**Background:**

High levels of future anxiety in parents may not only affect their cognitive processes, and attitudes but also impact their parenting behaviour and relations with the children.

**Objective:**

In the present study, we aimed to identify the differences in the intensity of parents’ future anxiety across demographic variables and to assess demographic predictors of FA.

**Methods:**

A group of 103 parents from Eastern Poland (71% mothers and 29% fathers) completed the Future Anxiety Scale. Their children were aged 3—16 years. The 10 aspects of Future Anxiety were compared across the five parent demographic variables of gender, age, level of education, place of living (rural vs. urban), and number of children.

**Results:**

The results show that parents’ education level, gender, and the number of children in the family were predictors of FA. Parents’ age and place of living did not significantly predict FA. Mothers reported significantly higher levels of anxiety than fathers, for both general future anxiety and helplessness. Parents who had only one child reported higher levels of helplessness. However, it was the parents’ level of education that showed the greatest number of significant differences. Parents with only secondary education reported higher average scores on seven (70%) of the 10 aspects of future anxiety. The differences were significant for general future anxiety, health and wellbeing, restricted freedom, the meaning of life, pessimism, helplessness, and isolation.

**Conclusion:**

These findings indicated the possible groups of parents who may need support and identified potential areas of intervention.

## Introduction

Excessive anxiety about the future (Future anxiety, FA) affects the cognitive processes, attitudes, and behaviour of the individual. It makes functioning difficult in the present, both in the personal area, as well as own and social activity (Zaleski, 2018). A high level of FA is associated with low wellbeing, and it consumes energy that should be devoted to overcoming challenges and impedes creative activity (Dadaczynski et al., 2021). It may result in passivity, novelty avoidance, disorganization in everyday life, and a decrease in future planning (Dodd et al., 2021). In recent years, due to the global COVID-19 pandemic, and consequently greater uncertainty about the health and economic situation, the proximity of armed conflicts and increased migration, researchers’ interest in studying FA has increased significantly. In this context, identifying groups of people exhibiting high levels of FA is important for introducing an array of effective countermeasures.

### Background of the FA concept

FA can be understood as a state of uncertainty, worry, precariousness, tension, and concern about unfavourable changes in a more remote personal future (Zaleski et al., 2017). The theoretical base of this concept is the motivational model of hope and fear developed by Zaleski (2018) at the end of the twentieth century, which explains that the positive cognitive representation of future events raises hope and helps to focus on activities that lead to completion of the goal. But a negative cognitive representation of future events results in anxiety about the future, and the individual may either implement preventive mechanisms, which may help them overcome fear and raise hope, or use immature defensive mechanisms and, consequently, experience increased fear and lose hope (Zaleski, 2018). According to previous literature, FA “refers to attitudes toward the future in which negative cognitive and emotional processes outweigh positive ones and in which fear is stronger than hope. It is a fear of future events and a feeling that dangerous or adverse changes may occur” (Zaleski, 1996, p. 167). The fear experienced is overt and conscious, and it is caused not by the actual events, but by cognitive representations of the future. FA contains two of the most important features of a pessimistic view of the future, (i.e., having dark expectations of what is forthcoming and, consequently, giving up quite easily on future plans) (Jannini et al., 2022).

Zaleski (2018) and Zaleski et al. (2017) also developed the Future Anxiety Scale (FAS) as an individual’s self-report measure of anxiety related to their perception of their future. There are five FAS questionnaires (FAS1 to FAS5), each having a different number of items, ranging from 56 to only five (Duplaga & Grysztar, 2021; Zaleski et al., 2017). They have been adapted in English, French, German, Turkish, Italian and Spanish and used in cross-cultural studies (Jannini et al., 2022; Torrado et al., 2024; Yıldırım et al., 2023). The period of the pandemic has meant that researchers have been focused on studying FA together with general anxiety, stress or depression, and with the sociodemographic predictors and features related to the course of anxiety about the COVID-19 pandemic (Duplaga & Grysztar, 2021).

### Predictors of FA

The intensity of FA depends on intrapersonal factors which include, among others, the subjective importance of the anticipated event, the estimated probability of its occurrence, sense of self-efficacy (Tucholska et al., 2021), and resilience, the latter of which is negatively correlated to FA (Mutia & Hargiana, 2021). Previous studies have also indicated some sociodemographic risk factors which possibly affect the severity of anxiety in adults. These are age, gender, level of knowledge, social and economic status including employment, marital status, and place of residence (Scott et al., 2010).

In general, female gender and younger age predicted higher anxiety, including FA (Dadaczynski et al., 2021; Jacobi et al., 2014; Jannini et al., 2022; Maske et al., 2016; McLean & Anderson, 2009; McLean et al., 2011). Students with lower social backgrounds, members of lower social classes and the unemployed have reported higher anxiety and FA (Alonso et al., 2004; Awad et al., 2024; Lorini et al., 2023; Pechmann et al., 2014). A study by Ta et al. (2017) reported that single people have a higher level of anxiety than married people. Place of residence might also be a factor for stress, with a higher risk for people who live in large urban areas than those who live in rural areas (Alonso et al., 2004). Also, higher level of education among adults is associated with less psychological distress (Grzywacz et al., 2004), and lower education is associated with increased risk of late life depression (Chang-Quan et al., 2010; Huang et al., 2010).

In the study by Duplaga and Grysztar (2021), conducted in the Polish population following the first phase of the COVID-19 pandemic lockdown, from sociodemographic variables, only gender, vocational status, and income were associated with the level of FA. A higher level of FA was found among women than men, persons with lower monthly incomes than average, and employees of the public or private sector than the self-employed or farmers. Interestingly, the level of FA was not associated with other factors such as place of residence, marital status, or level of education.

### Why parents are an essential group to study

Exploration of FA experience may be relevant in families, especially among parents who worry about creating the educational and social environment necessary for the proper development of their children. So far, the main focus of studies has been on parents of children with developmental disorders like Down syndrome, autism, and behavioural disorders (Bujnowska et al., 2019). FA in parents of children with typical development has not been studied. However, a review of studies related to general anxiety of parents and anxiety related with parenting may form the basis for consideration of FA in this group.

Research shows that a high level of anxiety in parents is associated with parenting behaviour and nurturing practices in several ways. Firstly, more anxious parents show less optimism, less productive engagement, and more withdrawn parenting behaviours with their children than less anxious parents (Woodruff-Borden et al., 2002). Mothers with higher levels of anxiety demonstrated intrusiveness, and a poorer quality of mother–child relationship (Dib et al., 2019), while fathers had lower consistency and higher hostility (Giallo et al., 2015). Secondly, high levels of anxiety may lead to changes in appraisal. Parents may view their child’s environment in accordance with their own negative perspective of the world or perceive the child’s behaviours as more negative or threatening (Lester et al., 2009). Thirdly, high anxiety may reduce maternal sensitivity to infant needs (Booth et al., 2018), reduce tolerance of children’s negative emotions (Creswell et al., 2013) or make parents less able to effectively read their child’s cues and respond to their child (Carreras et al., 2019). Lastly, a connection has been found between a parent’s anxious and excessive controlling behaviour towards their child and the increased risk of future anxiety in the child (Lawrence et al., 2019; Sydsjö et al., 2018).

With a high level of FA, tasks related to a child’s upbringing, building a relationship with the child, and creating a benevolent climate for the upbringing can be difficult for the parents. Then, such a state can be detrimental to the parents’ functioning and hence families as a system. Hence, it is crucial to identify parents who are at risk of experiencing a high level of FA. By identifying groups at a higher risk of developing FA, this study can inform policies that can be tailored to help them.

### Present study

The present study aimed to identify the differences in the intensity of FA across demographic variables (gender, age group, level of education, place of living, and number of children) among parents. Furthermore, we also assessed the extent to which demographic variables predicted FA. For this aim, we controlled for the shared variance of the demographic variables, which allowed us to assess the unique effects of each of them on FA. Identifying demographic variables associated with a high level of FA could assist psychologists, social workers, and family doctors to prevent or provide interventions aimed at reducing tension and the uncertainty connected with FA. Thereby improving parents’ and, by association, children’s quality of life at physiological, psychological, and social levels.

## Methods

### Participants and procedure

A total of 124 parents responded to the request to participate in the study but 21 (17%) parents did not meet the inclusion and exclusion criteria. The remaining 103 parents constitute the sample reported here. The parents were recruited through invitation letters distributed by directors of kindergartens and primary schools in Eastern Poland during the 2016/2017 school year. Inclusion criteria for parents were: (a) at least one child aged between 3 and 16 years of age; (b) the child was living with parents or one parent, and the exclusion criteria were: (a) suspected or confirmed diagnosis of developmental disabilities for the child; (b) missing responses to more than 5% of the questionnaire items.

Parents were contacted through randomly chosen preschools, primary and junior high schools in Eastern Poland. Invitations to participate were sent to the headmasters and included a description of the study. Participants completed the questionnaire in groups of up to 25, within a 30-min time frame in a designated room at the kindergartens and schools during the parent-teacher meetings, after the first semester of the school year. Before responding to the questionnaire, all participants received the Free and Informed Consent Form with Participant Information Sheet that informed them about the aim of the study, the procedure to be followed, and that participation was voluntary and anonymous, and that the results would only be used for scientific purposes. A guarantee about the ethical treatment of the data was also given. The parents were also given the option to discuss any issues with the researcher after completing the questionnaire. This study was conducted in accordance with The Code of Ethics of the World Medical Association (Declaration of Helsinki), which reflects the ethical principles for research involving humans (Williams, 2008). It was approved by the relevant Research Ethics Committee and all procedures followed relevant laws and institutional guidelines. The manuscript data is available upon request from the author.

### Measures

Participants completed a semi-structured questionnaire, which collected demographic information and included the Future Anxiety Scale (FAS; Zaleski, 1996).

The FAS is a self-report questionnaire that assess an individual’s anxiety level concerning future events (Bujnowska et al., 2019; Zaleski, 2018). The present study employed the original 38-item Polish version of the FAS. Items are formulated as negative or positive statements and respondents rated each item using a 7-point Likert scale ranging from 0 (*strongly disagree*) to 6 (*strongly agree*). The FAS provides 11 scores: general future anxiety level, catastrophe, health and wellbeing, restricted freedom, the meaning of life, politics and economy, achievements, pessimism, social relations, helplessness, and isolation. There is currently no normative data for the FAS, but higher scores indicate a higher level of FA. The test–retest reliability for 40 subjects after 35 days was 0.85 (Zaleski, 1996).

In the present sample, high internal consistency reliabilities (Cronbach alpha) were found for 10 of the 11 subscales: general future anxiety (α = 0.88), health and wellbeing (α = 0.71), restricted freedom (α = 0.79), the meaning of life (α = 0.82), politics and economy (α = 0.77), achievements (α = 0.89), pessimism (α = 0.81), social relations (α = 0.80), helplessness (α = 0.80), and isolation (α = 0.84). The reliability of the catastrophe subscale was unacceptably low (α = 0.45), so it was excluded from further statistical analysis.

### Data analysis

Preliminary examination of the data showed that the assumptions required for the use of parametric statistics (e.g., skewness and kurtosis) were met. All analyses were conducted using SPSS for Windows Version 24. Differences were considered significant at alpha level of *p* < 0.05. The 10 aspects of FA were compared across the five demographic variables of gender of parent, age group (20 – 40 vs. 41 + years), level of education (secondary vs. tertiary), place of living (rural vs. urban), and number of children (one child vs. more than one child). To examine for differences across demographic variables independent-samples *t*-tests were used and effect size (Cohen’s *d*) was interpreted as 0.20 = small, 0.50 = medium, and 0.80 = large (Cohen, 1988). The variables titled “relationship status” and “household employment” were not used to examine the differences across demographic variables due to two main reasons. Firstly, only 12 (12%) of the parents were not married and the vast majority (84%) of parents came from households where both parents were employed. Secondly, Multivariate Analysis of Variance showed that there were no statistically significant differences between single and married/couple parents (*p* = 0.202), and between employed and unemployed parents (*p* = 0.062).

To assess the extent to which demographic variables predicted FA a series of ten multiple linear regression models were conducted. In each model the five demographic variables (i.e., gender, age, place of living, level of education, and number of children) were entered as predictors of the specific aspect of FA.

## Results

### Distribution across socioeconomic and demographic factors

The participants were 73 (71%) mothers and 30 (29%) fathers, the majority of them were aged between 20 and 40 years old (68%), were married (88%), lived in large or medium-size cities (70%), had one child (52%), completed a University degree (71%) (Bachelor or Master´s), and both parents in family were in paid employed (84%). Demographic information is presented in Table 1.

**Table 1:** Demographic Characteristics of the Parents (*N* = 103)

Variable		*n* (%)
Gender	Female	73 (71%)
	Male	30 (29%)
Age	20—40 years	70 (68%)
	41—60 years	33 (32%)
Relationship status	Single	12 (12%)
	Married/couple	91 (88%)
Level of education	Secondary	30 (29%)
	Tertiary	73 (71%)
Employment	One or both unemployed	16 (16%)
	Both employed	87 (84%)
Place of living	Urban	72 (70%)
	Rural	31 (30%)
Number of children	One	54 (52%)
	Two or more	49 (48%)

### Descriptive statistics and intercorrelations between the study variables

The descriptive statistics and intercorrelations for all variables in the study are presented in Table 2. The relationships between the demographic variables were not significant, except for the relationship between age and gender. All of the FA scales were positively related one to another. There were several significant associations between the demographic variables and the FA scales; education especially seems to be associated with FA. Gender was negatively related to general FA and helplessness, while the number of children was negatively associated with helplessness. In turn, neither age nor the place of living was related to any of the FA subscales.

**Table 2:** Descriptive Statistics and the Zero-Order Correlations Between the Study Variables

	*M*	*SD*	1	2	3	4	5	6	7	8	9	10	11	12	13	14
Demographic variables																
1 Gender	1.29	0.46	-													
2 Age	1.32	0.47	**.29**													
3 Place of Living	1.70	0.46	-.14	-.09												
4 Education	1.71	0.46	.04	-.06	.09											
5 Number of children	1.48	0.50	.07	.05	-.10	-.12										
Future anxiety																
6. General	10.38	4.87	**-.20**	.13	-.05	**-.21**	-.06									
7. Health and well-being	9.35	3.91	-.01	.14	.02	**-.20**	-.03	**.72**								
8 Restricted freedom	7.45	3.66	-.08	.10	.04	**-.26**	-.06	**.81**	**.69**							
9 The meaning of life	7.07	3.85	-.08	.03	.03	**-.21**	-.03	**.80**	**.68**	**.86**						
10 Politics and economy	8.95	3.48	-.02	.08	-.02	-.12	-.04	**.72**	**.67**	**.62**	**.70**					
11 Achievements	11.28	4.72	.03	.12	-.03	-.13	-.14	**.78**	**.72**	**.73**	**.74**	**.82**				
12 Pessimism	7.71	3.59	-.08	.00	.01	**-.26**	-.05	**.81**	**.71**	**.77**	**.80**	**.78**	**.86**			
13 Social relations	12.03	4.58	-.18	.06	.08	-.14	-.05	**.73**	**.71**	**.68**	**.70**	**.74**	**.67**	**.72**		
14 Helplessness	7.95	3.67	**-.21**	.06	.07	**-.29**	**-.20**	**.80**	**.70**	**.81**	**.84**	**.67**	**.78**	**.84**	**.75**	
15 Isolation	8.92	4.84	-.09	.11	.05	**-.25**	-.11	**.80**	**.66**	**.87**	**.87**	**.69**	**.76**	**.75**	**.68**	**.86**

Correlation coefficients of ≥ .20 are significant at *p* < .05 (marked in bold)

### Group comparisons

#### Gender and age

For gender, of the 10 aspects of FA examined, statistically significant differences were found for two. Females (*M* = 11.00, *SD* = 5.02) reported significantly higher levels of general future anxiety than males (*M* = 8.87, *SD* = 4.20; *t* (101) = 2.05, *p* = 0.043), with a small effect size (Cohen’s d = 0.46). Also, females (*M* = 8.45, *SD* = 3.64) reported significantly higher levels of helplessness than males (*M* = 6.73, *SD* = 3.50; *t* (101) = 2.20, *p* = 0.030). Again, the magnitude of the effect size was small (Cohen’s d = 0.48).

With respect to age, parents in the older age group (40 + years) reported higher average anxiety than parents in the younger age groupon eight of the 10 aspects of FA. However, none of these differences between parent age group and scores on the FA scales were statistically significant.

#### Education level

The differences between the parent level of education groups were significant on seven of the 10 aspects of FA. In all instances the parents from the lower education group reported higher average levels of FA.

Statistically significant differences were found for general future anxiety (*p* = 0.033), health and wellbeing (*p* = 0.048), restricted freedom (*p* = 0.009), the meaning of life (*p* = 0.032), pessimism (*p* = 0.002), helplessness (*p* = 0.001), and isolation (*p* = 0.012) (Table 3).

**Table 3:** Descriptive Statistics, t-statistics, and Effect Size for Parent’s Education Level and Future Anxiety

	Parents’ Education Level					
Future anxiety	Secondary (*n* = 30)		Tertiary (*n* = 73)			
	*M*	*SD*	*M*	*SD*	*t* (df = 101)	*d*
General future anxiety	11.97	4.59	9.73	4.87	2.16*	.47
Health and wellbeing	10.53	3.56	8.86	3.97	2.00*	.44
Restricted freedom	8.90	3.08	6.85	3.74	2.66*	.60
The meaning of life	8.33	3.51	6.55	3.89	2.18*	.48
Politics and economy	9.60	2.85	8.68	3.69	1.22	
Achievements	12.23	4.22	10.89	4.88	1.32	
Pessimism	9.17	2.64	7.11	3.77	3.15*	.64
Social relations	13.00	3.54	11.63	4.91	1.39	
Helplessness	9.60	2.67	7.27	3.82	3.51*	.72
Isolation	10.77	4.01	8.16	4.97	2.55*	.58

^*^p < .05

The magnitude of the effect size for general future anxiety, health and wellbeing, and the meaning of life was small; while for restricted freedom, pessimism, helplessness, and isolation the effect size was medium (Table 3).

#### Place of residence

With respect to the parents’ residence groups, those living in urban environments reported higher average anxiety than those living in rural environments on eight of the 10 aspects of FA. However, none of these differences between rural and urban residence and scores on the FA subscales were statistically significant.

#### Number of children

Parents with one child reported significantly higher levels of helplessness (*M* = 8.63, *SD* = 3.37) than parents with more than one child (*M* = 7.20, *SD* = 3.87; *t* (101) = 2.00, *p* = 0.049). The magnitude of the effect size was small (Cohen’s d = 0.40). The differences on the other nine aspects of FA were not statistically significant.

### Demographic variables predicting future anxiety

To test whether demographic variables predicted FA, we conducted a series of 10 multiple linear regressions. In each model, we entered the five demographic variables (i.e., gender, age, place of living, education, and the number of children) as predictors of the specific aspect of FA (Table 4).

**Table 4:** Multiple linear regression analysis results for demographic variables predicting different aspects of FA

Variables	General FA	Health & Wellbeing	Restricted Freedom	Meaning of Life	Politics & Economy	Achievements	Pessimism	Social Relations	Helplessness	Isolation
Constant	16.924 (4.929***)	11.077 (3.882***)	10.934 (4.164***)	10.558 (3.751)	10.666 (4.124***)	14.697 (4.265***)	12.563 (4.846***)	14.809 (4.449***)	14.604 (5.865***)	13.934 (4.046***)
Gender	-.251 (-2.496*)	-.035 (-.335)	-.097 (-.949)	-.079 (-.759)	-.041 (-.383)	.015 (.145)	-.000 (-.617)	-.193 (-1.857)	-.214 (-2.214*)	-.100 (-0.984)
Age	.191 (1.914)	.146 (1.410)	.120 (1.180)	.042 (.404)	.088 (.838)	.117 (1.129)	.005 (.047)	.112 (1.085)	.121 (1.260)	.137 (1.354)
Place of living	-.056 (-.578)	.037 (.368)	.053 (.543)	.042 (.416)	-.007 (-.067)	-.018 (-.184)	.015 (.148)	.070 (.705)	.062 (.660)	.060 (0.615)
Education	-.194 (-2.007*)	-.194 (-1.947)	-.259 (-2.642**)	-.216 (-2.158*)	-.119 (-1.167)	-.141 (-1.412)	-.270 (-2.729**)	-.135 (-1.353)	-0.305 (-3.285***)	-.255 (-2.618**)
Number of children	-.082 (-.853)	-.055 (-.552)	-.082 (-.842)	-.050 (-.498)	-.053 (-.526)	-.170 (-1.700)	-.079 (-.796)	-.045 (-.456)	-0.215 (-2.322*)	-.138 (-1.417)
R_adj._^2^	.078	.013	.046	.008	-.026	.010	.032	.018	.146	.059
*F*(5.102)	2.737*	1.260	1.992	1.160	.486	1.199	1.682	1.378	4.483***	2.281

Values in the table are the β regression coefficient, and those in brackets are the Student *t.* * *p* = < 0.5, ** *p* = < 0.01, *** *p* ≤ 0.001

These results further supported the role of education, which was marked as a significant predictor of six aspects of FA (i.e., general, restricted freedom, the meaning of life, pessimism, helplessness, and isolation) even after accounting for the shared variance with the remaining demographic variables. In addition, gender was a significant predictor of general FA as well as helplessness, the latter of which was also predicted by the number of children. Neither age nor the place of residence were significant predictors of any aspect of FA.

## Discussion

The present study was designed to identify the differences in the intensity of 10 dimensions of FA for a group of parents across the five demographic variables of gender, age, level of education, place of living, and number of children. It also assessed the extent to which these demographic variables predict FA. The results showed that the level of FA is affected by three demographic factors: parents’ level of education and gender, and by the number of children in the family. Both, the group comparison model and the regression analyses supported these findings. These findings are consistent with previous findings suggesting that a lower level of education is associated with a higher level of general anxiety (Dodd et al., 2021; Grzywacz et al., 2004), and that women have a higher intensity of anxiety than men (Dadaczynski et al., 2021; Jannini et al., 2022; Torrado et al., 2024).

### Level of education

Parents’ level of education was a significant predictor for general anxiety about the future and six specific dimensions of FA: restricted freedom, the meaning of life, pessimism, helplessness, isolation, and health and wellbeing. In these aspects, parents with secondary education reported a higher intensity of FA than parents with a tertiary level of education. The strongest influence of a parents’ education level (medium effect) was reported for anxiety about the future in personal (*helplessness, pessimism)*, own activity (*restricted freedom)*, and social (*isolation)* areas.

In the personal area (*helplessness, pessimism)*, mothers and fathers with lower education perceived the future more pessimistically and reported less sense of their influence upon their personal situation. These findings may be the result of two factors. Firstly, the economic and social context of low education is linked with socioeconomical status (Hudson, 2005) which might influence emotional and physiological responses to daily hassles, and to chronic stressors such as the lack of material resources and financial stress (Lorant et al., 2003), all of which can lead to feelings of burden and increase risk for anxiety or depressive symptoms (Gallo, 2009). Secondly, a higher level of education is associated with greater psychosocial resources that, in the general population, lead to better management of life difficulties (Niemeyer et al., 2019) and, in the case of parents, lead to less parental anxiety about children’s current and future well-being (Nomaguchi & Brown, 2011).

In the own activity area (*restricted freedom*) parents with lower education reported more concerns about their effectiveness in overcoming difficulties and managing their own lives in the future, as well as the possibilities of accomplishing their life goals and making their own decisions. These findings are in line with the motivational model of hope and fear by Zaleski (2018), which explains that a high level of FA may negatively affect functioning not only on the cognitive level but also on the executive one. Thus, it can manifest itself in protective and preventive actions that aim to protect what one has; the reluctance to undertake new and creative, activities; and adhering to well-known ways and places, as well as employment of routine methods, to solve life problems (Zaleski, 1996). Although the actual events do not cause the anxiety experienced because they are future events, parents experience the mental and physiological reactions in the here and now. The FA absorbs their energy and influences current actions and decisions to reduce this uncomfortable tension (Duplaga & Grysztar, 2021).

Finally, the lower the parents’ education, the higher the reported FA in the social area (*isolation*). These results are consistent with other research, which found that mothers with a college or advanced degree reported less parenting anxiety than mothers with lower levels of education (Nomaguchi & Brown, 2011; Widarsson et al., 2014). Furthermore, low education promoted stress in mothers regarding social isolation and spouse relationship problems (Widarsson et al., 2014). Also, high education status can act as a buffer against the effects of stress in the family (Almeida et al., 2005; Grzywacz et al., 2004).

In addition, the results of this study demonstrated the smaller, but still statistically significant, influence of parental education level on *general future* anxiety and worries about *the meaning of life* and *health and wellbeing*. Parents with only secondary education were more focused on the fear of a sudden accident or illness that would lower their well-being or make them unable to take care of their family and result in them becoming a burden for their family in the future. Our results are consistent with prior findings that education is regarded as a socioeconomic factor that has the potential to affect individuals’ wellbeing (Patria, 2022; Ruiu & Ruiu, 2019) and health outcomes (Huong et al., 2017). In addition, education seems to serve as a resource for making meaning of one’s current experiences as well as providing a positive outlook for the future (Nikolaev, 2018). On theoretical level, the suggested link between education and wellbeing is grounded in the expansion of capabilities, the sense of choice and competency and a certain level of coherence of how the world works (Wilson & Lomas, 2024).

### Gender

Findings from the present study also indicated that gender affected, though with a small effect size, parents’ FA level. Mothers reported significantly higher levels of anxiety than fathers for both general future anxiety and helplessness. These findings are consistent with those from previous studies which have reported that women experience more anxiety than men (Dadaczynski et al., 2021; Guszkowska & Bodasińska, 2023; Jannini et al., 2022; Torrado et al., 2024). Moreover, women are more likely than men to appraise threatening events as more stressful and less controllable (McLean & Anderson, 2009; McLean et al., 2011). Women list concerns about family and health-related events more frequently than men, whereas men list relationship, finance, and work-related events (Möller et al., 2015). In general, women score significantly higher than men on somatic symptoms and psychological distress (Matud, 2004).

### Number of children in the family

Lastly, it was also found that the factor of number of children in the family was related to the intensity of mothers’ and fathers’ FA about helplessness. Parents raising one child reported more helplessness that parents with two or more children. This finding is in line with previous results, which have showed that mothers of one child, more often than mothers of two and more children, use in parallel both parenting styles: domination over the child and focusing on the child (Bochniarz, 2010). Hence these mothers, on the one hand, subordinate their child strictly from a position of power and without considering the child’s needs. On the other hand, these parents also are overparenting, overprotecting and over-caring for the child, which is associated with parental anxiety and tension (Nelson, 2010; Segrin et al., 2013). As a result, these parents show greater helplessness in the face of problems associated with raising their only child (Bochniarz, 2010).

## Conclusion

These findings have shed light on the demographic factors that influence FA among parents. The demographic factors identified included education level, parent’s gender, and the number of children. Lower education, being female, and having only one child emerged as significant predictors of heightened helplessness. Additionally, lower education and being female were associated with increased general FA. Lower education also was associated with feelings of restricted freedom, isolation, pessimism, questioning life’s meaning, and compromised health and well-being.

From the results of this study it is clear that mothers with only one child and education below tertiary level are the highest-risk group for experiencing elevated FA. For parents with lower education levels, irrespective of whether they were males or females, increased FA had significant challenges including negative effects on well-being, daily activities, social interactions, and health. From a psychological perspective, heightened levels of helplessness and general FA can impede the ability to effectively fulfill parental roles. The results of this study can provide a starting point for investigating the link between the intensity of parents’ FA and their effectiveness in the parental role.

These findings may also pave the way for developing parental support services dedicated to parents of children without any special needs. Most of the existing parent support programs are devoted to parents of children with developmental disability, behavioural issues, or other difficulties. Our findings indicated the possible groups of parents with children who don’t have special needs, but who may still need support, as well as the potential areas of interventions for such parents. Implementing interventions based on Zaleski’s (1996) motivation of fear and hope theory could alleviate anxiety and bolster hope in these parents. Elevated levels of hope are linked to positive psychosocial outcomes, adaptive coping strategies, reduced depression and anxiety (Snyder, 2002), and play a pivotal role in therapeutic change processes (Ward & Wampler, 2010). Finally, this study was conducted in the EU region which is adjacent to Ukraine and was completed before the COVID-19 pandemic and the onset of the war in Ukraine. Given this context, this study’s results serve as a valuable baseline for FA researchers to investigate parental functioning in the face of conflicts.

### Future directions and limitations

While the findings presented in this study are potentially useful, several limitations need to be acknowledged. One of the primary limitations is that the sample was only of parents from Eastern Poland. This could hinder the generalizability of the findings to broader populations with different cultural or geographical backgrounds. Future research should aim to include a larger and more internationally diverse sample of parents. Additionally, this study employed a cross-sectional design which, while it allowed for the observation of associations between variables, cannot be used to establish causality. In the future, longitudinal studies should be undertaken as they would offer a more comprehensive understanding of changes in FA over time and could potentially identify causal relationships between demographic factors and FA. Another limitation of our study is that we focused exclusively on sociodemographic variables related to parents, without considering child-related variables such as the child’s age. These factors, particularly age, may influence parents future anxiety levels due to different developmental stages and the evolving demands placed on parents. Future studies could provide a more comprehensive understanding of FA by incorporating child-specific factors and examining how parental FA levels differ across the child’s developmental stages. It would also be interesting and useful to revisit parent’s level of FA in the same region, not only post-pandemic but also during the current times of heightened conflict and hostilities resulting from Russia’s invasion of Ukraine. While recent studies suggest an increase in FA post-pandemic (Dadaczynski et al., 2021; Dodd et al., 2021; Duplaga & Grysztar, 2021), there is limited research that focuses on parents specifically. Exploring parents’ FA in these challenging contexts could provide a deeper understanding of its dynamics and the factors contributing to it.

## Data Availability

Data will be made available on request.

## Abbreviations

FA: Future anxiety
FAS: Future Anxiety Scale
